# The Role of HCN Channels on Membrane Excitability in the Nervous System

**DOI:** 10.1155/2012/619747

**Published:** 2012-08-13

**Authors:** Daisuke Kase, Keiji Imoto

**Affiliations:** ^1^Department of Information Physiology, National Institute for Physiological Sciences, Myodaiji, Okazaki 444-8787, Japan; ^2^School of Life Sciences, The Graduate University for Advanced Studies, Myodaiji, Okazaki 444-8787, Japan

## Abstract

Hyperpolarization-activated and cyclic nucleotide-gated (HCN) channels were first reported in heart cells and are recently known to be involved in a variety of neural functions in healthy and diseased brains. HCN channels generate inward currents when the membrane potential is hyperpolarized. Voltage dependence of HCN channels is regulated by intracellular signaling cascades, which contain cyclic AMP, PIP_2_, and TRIP8b. In addition, voltage-gated potassium channels have a strong influence on HCN channel activity. Because of these funny features, HCN channel currents, previously called funny currents, can have a wide range of functions that are determined by a delicate balance of modulatory factors. These multifaceted features also make it difficult to predict and elucidate the functional role of HCN channels in actual neurons. In this paper, we focus on the impacts of HCN channels on neural activity. The functions of HCN channels reported previously will be summarized, and their mechanisms will be explained by using numerical simulation of simplified model neurons.

## 1. Introduction

Hyperpolarization-activated and cyclic nucleotide-gated (HCN) channels, first identified in 1976 in the heart by Noma and Irisawa [1] and characterized by Brown and Difrancesco [2] and Weiss and his colleague [3], are cation channels that open when the membrane potential is hyperpolarized. The general structure of HCN channels resembles that of the voltage-gated K^+^ channels. HCN channels consist of four subunits that have six transmembrane segments [4], the canonical GYG sequence in the pore forming region, and the positively charged S4 segment [4, 5]. But the K^+^ permeability of HCN channels is not so selective as typical K^+^ channels and is permeable to Na^+^ [6]. Thus a typical current reversal potential of HCN channels is around −30 mV. For voltage-dependent gating, inward movement of S4 segment in response to hyperpolarization is reported [7–9], but molecular aspects of channel opening are still unknown. For example, HCN channels require extracellular Cl^−^ and extracellular K^+^ to open [4]. Cyclic-AMP-(cAMP-) binding site locates near the C terminus, and cAMP affects the voltage dependence of activation in some HCN channel isoforms [10]. Phosphatidylinositol 4,5-bisphosphate (PIP_2_) is also known as a modulator of HCN channels; it shifts the voltage dependence through a different mechanism from that of cAMP [11, 12]. These multifaceted features endow HCN channels to work in many functions described below. In this paper, we try to understand the physiological significance of these functions by using simple numerical simulations.

## 2. Expression Pattern

Previous literatures reported that HCN channels are expressed in heart [1], brain [14], taste buds [15], and pancreatic cells [16]. This paper focuses on the electrophysiological function of HCN channels in the central nervous system. In the brain, HCN1 isoform is expressed strongly in cerebral cortex, hippocampus, and superior colliculus [17], and HCN2 is expressed ubiquitously in the brain. Expression of HCN3 and HCN4 is found in localized regions in the brain [18]. In the neuron, HCN channels are found in dendrites, somas, and axon terminals. Expression in axon terminals is reported at the calyx of Held [19] and the globus pallidus neurons [20]. Previous reports showed that expression of HCN channels on the cell surface is uneven in some neurons. HCN channels are expressed more strongly in distal dendrites than in somas in thalamic reticular neurons, whereas they distribute nearly evenly in thalamocortical relay neurons [21]. Recently, isoform-specific localization is also reported. For example, HCN1 is expressed strongly in dendrites in layer 5 pyramidal neurons of matured rats, whereas HCN2 is mainly expressed in the soma [22].

 Expression and kinetics of HCN channels are affected by unusual neuronal activity like epilepsy and environmental stimuli. Decrease in HCN channel expression is often observed in epilepsy models [23–26]. The molecular mechanism underlying HCN channel downregulation following epilepsy is not well understood. Recent studies showed that channel current and surface expression of HCN channels decrease in one hour after pilocarpine-induced status epilepticus, but decreases in total channel protein and mRNA occur in the subsequent week [27]. Altered voltage dependence is also reported in epilepsy. Involvement of activation of calcineurin and inactivation of P38 mitogen-activated protein kinase (p38 MAPK) in the hyperpolarizing shift of voltage-dependence is reported [25].

 On the other hand, some reports showed that sensory inputs are necessary to increase or keep the expression level of HCN channels. Deprivation of whiskers decreases the expression of dendritic HCN channels in the rat somatosensory cortex [28]. Interestingly, Adams et al. [24] reported increased HCN2 in CA3 and decreased HCN1 and HCN2 in CA1 in the same epilepsy model. Mechanisms underlying the regulation of HCN channel expression require further investigations.

## 3. Functional Roles of HCN Channels

**Figure 1:**
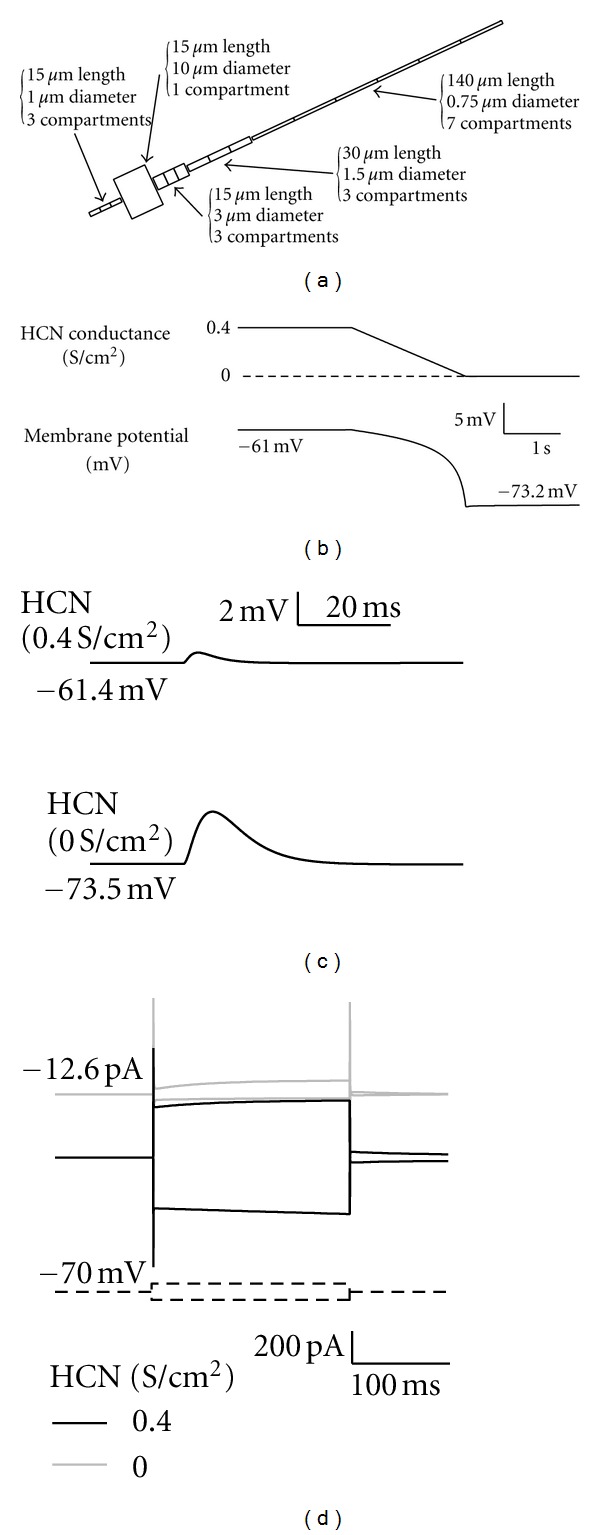
Simulation model and basic functions of HCN channels. (a) Structure of the simulated neuron [13]. EPSPs are measured at the soma. (b) The effect of HCN channels on membrane potential. Membrane potential becomes hyperpolarized (bottom) when HCN channel conductance decreases (top). (c) Excitatory effect of HCN channels. EPSPs are simulated using the model neuron with and without HCN channels. Because of the depolarized resting membrane potential, the membrane potential at the EPSP peak is more depolarized in the neuron with HCN channels than the neuron without HCN channels, although the EPSP amplitude of the neuron with HCN channels is much smaller. (d) The effect of HCN channels on membrane resistance is simulated in a voltage-clamp condition. Step pulses of ±10 mV require larger holding currents in the neuron with HCN channels (black) than the neuron without HCN channels (gray).

Based on the multifaceted features, HCN channels are involved in many kinds of functions that range from cellular to behavior levels (working memory task [29], initiation of neuropathic pain [10]). In this paper, we focus on neuron-level functions, which include regulation of resting membrane potential, normalization of synaptic inputs, selective filtering for coincident inputs, modulation of intrinsic cellular frequency characteristics, and regulation of membrane resistance. They are categorized into three groups: excitatory, inhibitory, and modulatory functions. We summarize these functions and show typical examples of stimulations obtained by using NEURON simulator (Figure 1(a)) [30]. Conditions for the simulation are summarized at the end of the text.

### 3.1. Excitatory Function

**Figure 2:**
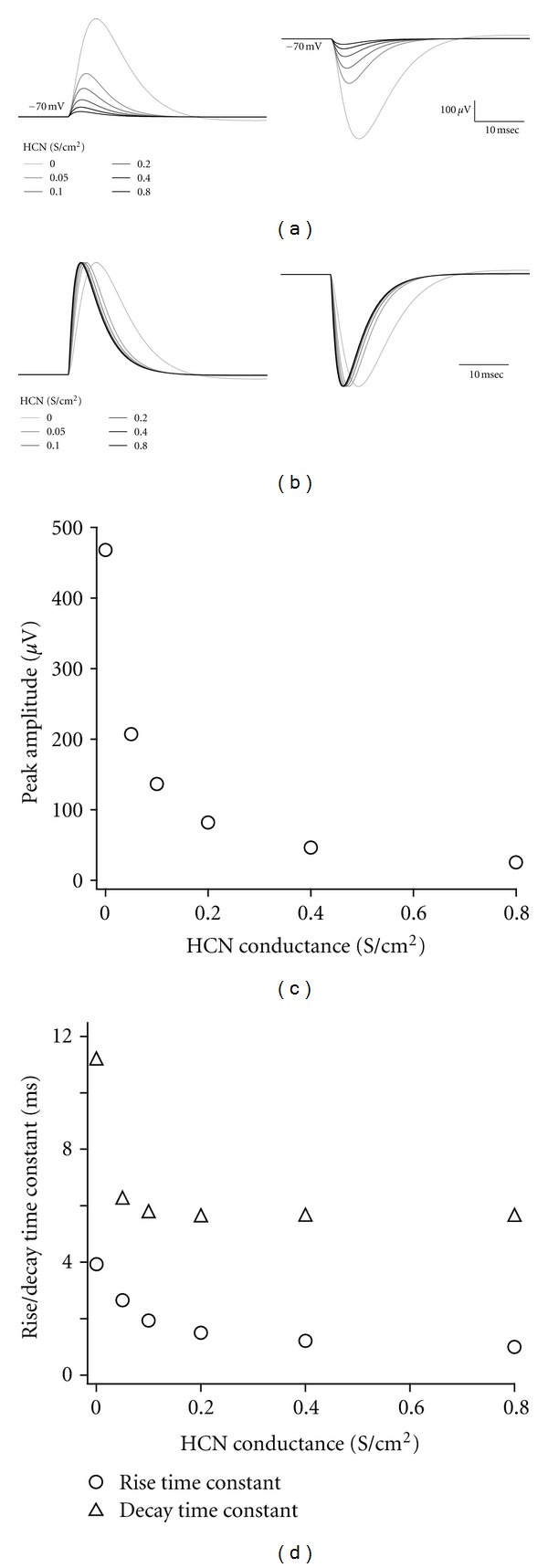
Inhibitory function of HCN channels. (a) Typical examples of EPSPs (left) and IPSPs (right) in the neuron with different HCN channel conductances. The initial membrane potentials are set to −70 mV by injecting currents. Synaptic input of the same intensity is applied. Color of each trace indicates the corresponding HCN channel conductance shown in the inset. (b) Normalized EPSP and IPSP traces shown in (a). (c) Relationship between the peak EPSP amplitude and HCN channel conductance. Even a small HCN channel conductance causes a dramatic reduction of the EPSP amplitude. (d) Relationship between rise (open circles) or decay (open triangles) time constants of EPSPs and HCN channel conductance. Time constants are obtained by single-exponential fitting. Both rise and decay time constants become shortened when HCN channel conductance increases. Asymptotic values of time constants are strongly dependent on the parameters of artificial synapse.

Because a typical current reversal potential of HCN channels is about −30 mV that is higher than the normal threshold for the generation of action potential, HCN channel currents depolarize and control the membrane potential near the resting membrane potential. When HCN channel conductance is removed, a simulated neuron shows a hyperpolarized membrane potential (Figure 1(b)). For example, blockade of HCN channels induces hyperpolarization in the rat superior colliculus [17]. Although HCN channel conductance decreases the EPSP amplitude (see below and Figure 2(a)), this inhibitory effect is masked by depolarization of the membrane potential (Figure 1(c)). In addition, the effect of inhibitory synaptic inputs is suppressed by HCN channels, because hyperpolarizing conductance of inhibitory synaptic input opens HCN channels to evoke depolarizing conductance [31].

 When voltage dependence of HCN channels is shifted to the depolarizing direction by cAMP or PIP_2_, HCN channels can be active at around the resting membrane potential and can work as an excitatory factor. Furthermore, in an interesting case, dendritic HCN channels are necessary to generate dendritic spikes at a short and fixed latency following optic fiber stimulation in the rat superior colliculus [17].

### 3.2. Inhibitory Function

Activated HCN channel conductance reduces the membrane resistance (Figure 1(d)). This reduction suppresses the impact of synaptic inputs on membrane potential. As HCN channels activate more, the amplitude and kinetics of postsynaptic potentials become smaller and shorter (Figures 2(a), 2(b), 2(c), and 2(d)).

### 3.3. Modulatory Function

**Figure 3:**
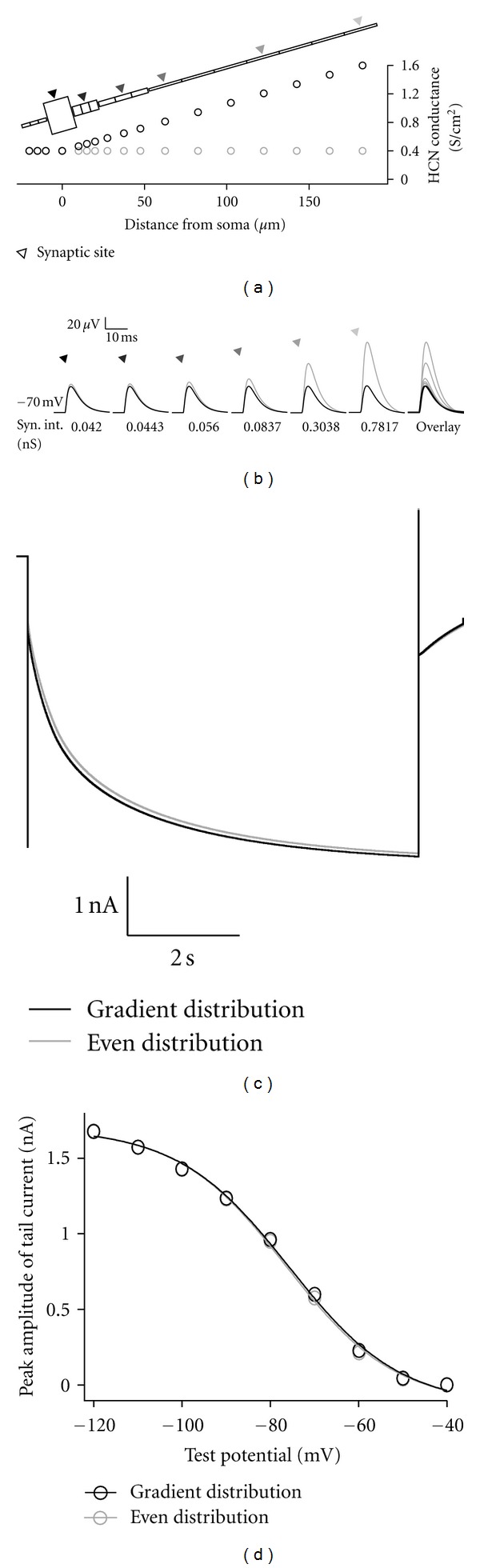
Gradient distribution of HCN channel conductance scales the impact of synaptic inputs. (a) Distribution of HCN channel conductance. In the gradient distribution model, density of HCN channel conductance increases 4 times in 200 *μ*m. Triangles shown on the dendrite and the soma indicate synaptic sites. (b) Typical somatic EPSPs in response to synaptic input to each indicated site in the gradient model (black) and in the even distribution model (gray). Color of each triangle corresponds to the synaptic site with the same color in (a). Rightmost trace shows superimposed traces. For each synaptic site, intensity of synaptic input is adjusted in the gradient model to obtain similar somatic EPSP amplitude, and the input of the same intensity is applied to the corresponding site of the even distribution model. (c) Typical traces of HCN channel currents in the gradient (black) and even distribution (gray) models. Membrane potential is stepped from −60 mV to −120 mV, held for 7 sec to activate HCN channels, and stepped to −80 mV to record the tail currents. (d) Voltage dependence of tail current amplitude of HCN channels in the gradient (black) and even distribution (gray) models. Both current-voltage relations overlap well in this condition.

Dendritic HCN channels normalize synaptic inputs in the cortical and hippocampal neurons. Current density of HCN channels increases along with the distance from the soma in hippocampal [32] and neocortical pyramidal neurons [33]. Such dendritic HCN channels scale the EPSP amplitude measured at the soma and suppress the location dependency of synaptic inputs in hippocampal [34] and neocortical pyramidal neurons [35]. We use two models with the gradient and even HCN channel distributions (Figure 3(a)), and computer simulation can reproduce a similar effect (Figure 3(b)). Interestingly note that, despite of different HCN channel distributions, both models show similar current amplitudes and overlapping voltage dependence of HCN channels in this condition (Figures 3(c) and 3(d)). 

**Figure 4:**
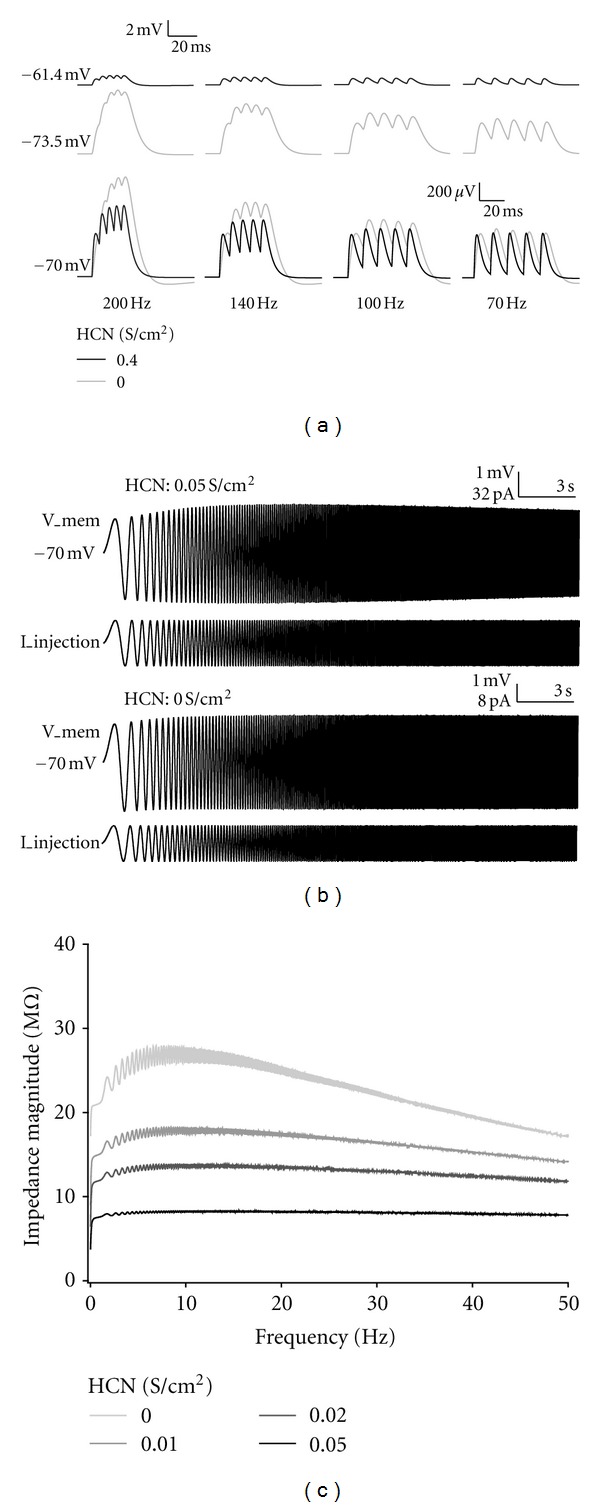
HCN channel conductance functions as high-pass filter to both synaptic inputs and intrinsic activities. (a) Examples of EPSPs in response to repetitive inputs at different frequencies in the models with (black) and without (gray) HCN channels. Membrane potential is not modified by current injection, and intensity of synaptic inputs is 0.4 nS for each HCN channel density (top). Intensities of synaptic inputs are set to obtain similar EPSP peak amplitudes evoked by single stimulation (0.42 nS for 0.4 S/cm^2^ of HCN channel, 0.043 nS for 0 S/cm^2^ of HCN channel) (bottom). The initial membrane potential is adjusted to −70 mV by current injection. (b) Typical examples of voltage responses (upper column) to sinusoidal current injection (lower column) in models with (top) and without (bottom) HCN channels. Initial membrane potential is set to −70 mV by current injection. Sinusoidal current is injected into the soma. Frequency of the sinusoidal current is increased from 0.1 to 50 Hz in 50 sec. Membrane potential and sinusoidal current in the first 25 sec are shown in this panel. Amplitudes of the injected currents are adjusted to obtain membrane potential changes of similar amplitude. (c) The relation between impedance magnitude and frequency of sinusoidal currents in models with several HCN channel conductances. HCN channel conductance inserts a high-pass filter. Impedance magnitude is obtained by dividing the magnitude component of Fourier-transformed voltage responses by the magnitude component of Fourier-transformed sinusoidal current.

 Activated HCN channels are involved in the detection of coincidental synaptic inputs. Because HCN channel conductance shortens the rise time and decay time of the EPSPs through the decrease in membrane resistance (Figure 2), summation of repetitive EPSPs that occur at a low frequency is suppressed. This suppression filters out low-frequency inputs, and thus improves the selectivity for synchronous synaptic inputs [36] (Figure 4(a)).

 Active conductance of ion channels enables neurons to produce intrinsic membrane potential oscillation and resonance [37]. Such rhythmic activity can filter inputs at certain frequencies [38], and can influence the precision of spike timing [39, 40]. HCN channel currents filter out the slow frequency inputs [41, 42]. Our simulation successfully shows that HCN channel conductance suppresses intrinsic activities particularly around 10 Hz (Figures 4(b) and 4(c)).

## 4. Influence of Other Ion Channels on the Roles of HCN Channels

Because HCN channels can work as both excitatory and inhibitory factors, it is difficult to predict which is the major role of HCN channels in controlling neural activity without conducting actual experiments. Other ion channels that are activated at similar potentials make the prediction more difficult. For example, K^+^ channels and Ca_V_3 (T-type) Ca^2+^ channels can indirectly affect the activation of HCN channels through modulation of membrane potential and resistance, and vice versa [43, 44]. Especially, delayed-rectifier M-type K^+^ channels (Km), also called K_V_7 or KCNQ channels, have a large impact on the role of the HCN channels, because Km channels open slowly at relatively polarized potentials from about −60 mV and do not inactivate [45]. George et al. [44](2009) showed that Km channels determine whether HCN channels function as an excitatory or an inhibitory factor, on the basis of recordings from hippocampal pyramidal neurons and computational simulation of model neurons. 

**Figure 5:**
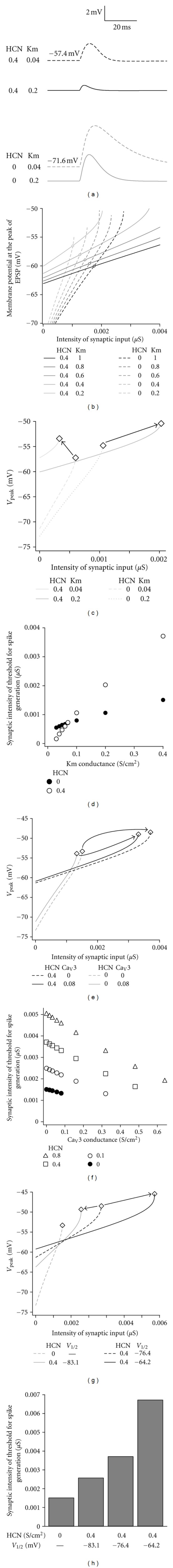
The roles of HCN channels are influenced by K^+^ channel and Ca^2+^ channel, and also modulated by voltage dependence. Values of channel conductance density (S/cm^2^) are shown in insets. (a) Typical EPSP traces in models with different HCN and muscarinic K^+^ (Km) channel conductances. Synaptic input of the same intensity is applied in the soma in this simulation. The initial membrane potential is not modified by current injection. (b) The relationship between membrane potential at the peak of EPSP (*V*_peak_) and intensity of synaptic input in models with different HCN and Km channel conductances. The *V*_peak_-intensity curves with and without HCN channel conductance cross. The cross points shift in a Km channel conductance-dependent manner. (c) Relation between *V*_peak_ and intensity of synaptic input in models with different HCN and Km channel conductances. Open diamonds indicate the threshold intensities for spike generation. The effect of adding HCN channel conductance is shown by arrows. Direction of the arrows is dependent on Km channel conductance. (d) Relationship between the minimum intensity of synaptic input to generate action potential and Km channel conductance in models with (open circles) and without (filled circles) HCN channels. When the Km channel conductance is small, the minimum intensity is smaller in the model with HCN channels than the model without HCN channels. In such a range of Km channel conductance, the role of HCN channels is excitatory. In contrast, when Km channel conductance is large, the main role of HCN channels is inhibitory. (e) Relation between *V*_peak_ and intensity of synaptic input in models with different HCN and Ca_V_3 channel conductances. Ca_V_3 conductance shifts the relation to the excitatory direction (to the left), whereas Km channel conductance shifts it to the inhibitory direction (to the right). (c) The influence of Ca_V_3 channel conductance appears milder than that of Km channel conductance. The effect of adding HCN channel conductance is shown by arrows. Direction of the arrows is independent of Ca_V_3 conductance. (f) Relationship between the minimum intensity of synaptic input to generate action potential and Ca_V_3 channel conductance in models with different HCN channel conductance. HCN channel conductance always functions as an inhibitory factor for action potential generation in out simulation, because condition with lower HCN channel density resulted in lower minimum stimulus intensity in all Ca_V_3 conductance. (g) Relationship between *V*_peak_ and intensity of synaptic input in models with different HCN channel conductance and voltage dependence. Open diamonds indicate the threshold intensities of synaptic input to generate action potential. Hyperpolarization and depolarization of voltage dependence, respectively, shift the relation to excitatory and inhibitory directions. These effects are similar to the modulation of HCN channel conductance. (h) Minimum intensity of synaptic input to generate action potential in models with different voltage dependencies of HCN channels.

 We further advanced the simulation study and examined the role of Km channel conductance on the threshold potential for spike generation by using a model neuron with conductances for HCN, Km, delayed rectifier K^+^, Na^+^, and Ca^2+^ channels. Our simulation reproduces a similar effect of Km channels on the HCN channel function (Figures 5(a) and 5(b)). For a weak synaptic input, the membrane potential at the peak EPSPs (*V*_peak_) is more depolarized with HCN channel conductance than without HCN channel conductance (Figure 5(b)). But as the synaptic input becomes stronger, the relation between *V*_peak_ with and without HCN channel conductance reverses. The crossing point of the relationship shifts in a Km conductance-dependent manner (Figure 5(b)). Because our model contains Na^+^ conductances, we can also analyze the effect of HCN channel conductance on the threshold potential to generate action potential in various Km conductance conditions. Our simulation suggests that when Km conductance is very small, HCN channel conductance reduces the minimum intensity of synaptic input to generate action potential. But when Km conductance becomes large, HCN channel conductance increases the minimum intensity of synaptic input to generate action potential (Figure 5(c)). The relationships between required intensity of synaptic input and Km conductance with and without HCN channel conductance crosses (Figure 5(d)). Conversely, when T-type Ca^2+^ channel conductance was altered to shift the action potential threshold, the relation of the threshold does not reverse in our simulation condition (Figures 5(e) and 5(f)). 

 A previous study showed that presynaptic HCN1 channels regulate Ca_V_3.2 channel activity and neurotransmission at specific cortical synapses [46]. In order to uncover how HCN channels contribute to the neural activity, it will be necessary to compare the effects of K^+^ channels and Ca^2+^ channels by actual experiments.

## 5. Effects of Modulators of HCN Channel

To elucidate the role of HCN channels in the neuron, modulators of HCN channels are indispensable. A number of modulators are reported for HCN channels (summarized by Lewis et al. [47]). Previous studies showed that cAMP, PIP_2_, and ethanol shift the voltage dependence to the depolarizing direction and accelerate the gating kinetics [48, 49]. In contrast, TRIP8b shifts the voltage dependence to the hyperpolarizing direction and slows the kinetics. These modulators can shift the voltage dependence by as large as 10 mV in either direction. We examined the effect of such shifts of voltage dependence on neural activity as well as effects of Km and T-type channel conductance. Briefly, hyperpolarizing and depolarizing shifts of the voltage dependence, respectively, result in decrease and increase in channel conductance (Figures 5(g) and 5(h)). Such modulation occurs during the maturation process in medial superior olive neurons [12]. Importance of modulated voltage dependence of HCN channels in the nervous system has already reported. For example, HCN channels are inhibited via suppression of cAMP by the activation of *α*2A-adrenoceptors that are colocalized with HCN channels in dendritic spines in the prefrontal cortex. This inhibition results in improved working memory in monkey and rat [29]. Increased neural activity caused by enhanced HCN channel activity with cAMP is involved in the initiation of neuropathic pain [10].

## 6. Involvement of HCN Channels in Neural Diseases

Previous reports showed the involvement of HCN channels in brain disorders, including absence seizures, febrile seizures, and Parkinson's disease. Reduced HCN channel activity following the establishment of status epilepticus is reported in many epilepsy models [23–26]. Decreased HCN channel activity is also reported in the external segment of the globus pallidus in rodent models of Parkinson's disease [50]. It is not known well, however, whether such reductions precede or follow the onset of the diseases. Genetic mutations of HCN channels are found in human epilepsy ([51, 52], and references therein). HCN2 null mice show absence epilepsy [53], and HCN1 null mice are more susceptible to kainic acid-induced seizures [13]. These reports imply, at least, dysfunction of HCN channel can promote the onset of seizures. 

## 7. Conclusion

Because HCN channels can work both excitatory and inhibitory, and these effects can be altered by pharmacological modulation, HCN channels are one of the promising pharmacological targets in treating diseases of the brain. Further studies about complex relations between HCN channels and other ion channels and about mechanisms underlying modulation of voltage dependence will provide us a concrete step for clinical usage of HCN channel modulation.

## 8. Simulation Condition

For computer simulation, a simplified neuron model is used, because the complicated dendritic structure can be compactified according to the Rall's rule [54] (Figure 1(a)). Membrane capacitance (1.4 *μ*F/cm^2^) and axial resistance (150 Ω*·*cm) are even throughout the neuronal structure. The model neuron contains conductances for two kinds of Na^+^ channels, delayed rectifier K^+^ channel, muscarinic K^+^ (Km) channel, and HCN channel. In addition, nonspecific leak conductance is set in all models (conductance, 40 *μ*S/cm^2^; reversal potential, −70 mV). Conductance for the T-type Ca^2+^ channel is included in some of the simulations, where addition is explicitly stated. Most conductances, except for delayed rectifier K^+^ and Km channels [55], are taken from a model of the rat subthalamic nucleus [56]. In order to change the voltage dependence, we shifted membrane potential ±7 mV in PROCEDURE section of mod file. Conductance density for each channel is summarized in Table 1. Unless stated otherwise, synaptic input is always applied to the soma using the Exp2Syn function of the NEURON simulator (tau1, 1.5 ms; tau2, 4 ms). Reversal potentials of excitatory and inhibitory inputs are 0 and −85 mV, respectively.

## Figures and Tables

**Table 1:** Parameters of ion channels in the neuron model.

Name of ion channel	Conductance (S/cm^2^)	Accession of ModelDB
Na^+^ channel	Soma: 0.1 dendrite: 0.01	74298
Original Na^+^ channel of Na^+^ channel	Initial segment: 0.1	Shift voltage dependence
Delayed rectifier K^+^ channel	0.4	101629
Muscarinic K^+^ channel	0.4	101629
HCN channel	0.4 (otherwise noted)	74298
Ca_v_3 Ca^2+^ channel	0.1	74298

ModelDB: https://senselab.med.yale.edu/modeldb.
